# Investigating Cell-ECM Contact Changes in Response to Hypoosmotic Stimulation of Hepatocytes In Vivo with DW-RICM

**DOI:** 10.1371/journal.pone.0048100

**Published:** 2012-10-26

**Authors:** Tabea A. Mundinger, Annika Sommerfeld, Roland Reinehr, Joachim P. Spatz, Dieter Häussinger, Heike Boehm

**Affiliations:** 1 Department of New Materials and Biosystems, Max Planck Institute for Intelligent Systems, Stuttgart, Germany; and Department of Biophysical Chemistry, University of Heidelberg, Heidelberg, Germany; 2 Clinic for Gastroenterology, Hepatology and Infectious Diseases, Heinrich-Heine-University Düsseldorf, Düsseldorf, Germany; National Center for Scientific Research Demokritos, Greece

## Abstract

Hepatocyte volume regulation has been shown to play an important role in cellular metabolism, proliferation, viability and especially in hepatic functions such as bile formation and proteolysis. Recent studies on liver explants led to the assumption that cell volume changes present a trigger for outside-in signaling via integrins, a protein family involved in mediating cellular response to binding to the extracellular matrix (ECM). However, it remains elusive how these volume change related signaling events are transducted on a single cell level and how these events are influenced and controlled by ECM interactions. One could speculate that an increase in cell volume leads to an increase in integrin/ECM contacts which causes activation of integrins, which act as mechano-sensors. In order to test this idea, it was an important issue to quantify the cell volume-dependence of the contact areas between the cell and the surrounding ECM. In this study we used two wavelength reflection interference contrast microscopy (DW-RICM) to directly observe the dynamics of cell-substrate contacts, mimicking cell-ECM interactions, in response to a controlled and well-defined volume change induced by hypoosmotic stimulation. This is the first time a non-invasive, label-free method is used to uncover a volume change related response of *in vitro* hepatocytes in real time. The cell cluster analysis we present here agrees well with previous studies on *ex vivo* whole liver explants. Moreover, we show that the increase in contact area after cell swelling is a reversible process, while the reorganisation of contacts depends on the type of ECM molecules presented to the cells. As our method complements common whole liver studies providing additional insight on a cell cluster level, we expect this technique to be particular suitable for further detailed studies of osmotic stimulation not only in hepatocytes, but also other cell types.

## Introduction

Cellular signaling is a crucial part of cell biology involving intra-, inter- or extracellular communication with an abundance of physical and chemical cues. In addition to a variety of local signaling mechanisms, including specific molecules carrying information or external forces, the entire cellular volume itself is employed by the cell as a crucial parameter in perceiving its environment. Fluctuations in cellular volume regulated by cellular hydration have been shown to serve as a potent signaling mechanism influencing cellular metabolism, proliferation and survival [1]–[3].

A common cause and mechanism for variations in cell volume and cell hydration is a change in the ambient osmolarity. Hence, the capability of cells to recognize fine fluctuations in the water content is referred to as osmosensing. Although cellular volume regulation plays an important role for most animal cells, a prominent example for a physiological system in which cellular hydration levels play an important role is found in the liver. Here, hepatocytes increase their volume as a result of hypoosmotic conditions, cumulative amino acid uptake or insulin stimulation and many signaling cascades crucial for liver function have been shown to be connected to volume regulation [4]. Studies on perfused rat liver and rat hepatocytes in culture have indicated the influence of osmosensing on a variety of biological processes. These processes include regulation of gene expression [5]–[8] as well as modulation of many cellular functions, such as carbohydrate and protein metabolism, inhibition of proteolysis, and bile formation [4], [9], [10]. Most importantly, experimental studies have revealed the crucial role of integrins, in particular RGD-peptide sensitive α5β1 integrins, in osmosensing [9], [11]–[14]. Furthermore, staining experiments showed the presence of activated β1 integrins immediately following hypoosmotic experiments [13], [15] This class of adhesion molecules has already been shown to exhibit outside-in as well as inside-out signaling [16]. As a result, these findings have led to the proposal that activation of specific integrins can act as a mediator in osmosensing mechanisms, coupling cellular volume changes to intra-cellular signaling pathways.

However, as these integrins are mainly involved in the signaling between ECM and the cell, ECM composition should have a critical impact on the integrin mediated osmosensing. Indeed, it has been shown, that in the absence of this integrin-ECM signaling in hepatocyte suspensions, proteolysis inhibition will not occur upon hypoosmotic or insulin stimulation [17]. These observations emphasize the importance of the ECM environment and the contact formation between ECM and cells for integrin signaling and thus for osmosignaling.

In addition to receptor signaling, the ECM also provides various other cues to the cells. The macromolecular building blocks of the ECM, such as collagen, laminin, or heparin sulfates, have the ability to bind and release a great amount of ions and water and thus regulate the osmotic microenvironment. Moreover, the ECM is highly sensitive to changes in the osmolarity and behaves quite distinctly under different osmotic conditions [18]. These variations are reflected in the permeability and the stiffness of the ECM, which add another level of complexity to the interaction of osmosensing and integrin-mediated signaling via cell-ECM contact.

Whole-liver explant experiments have provided valuable insight into osmosensing, mechanisms and the involvement of integrins [9], [12], [14]. However, to elucidate and, in particular, to quantify the interaction of cellular-ECM contact and osmosensing whole tissue studies are not suitable due to the intrinsic high complexity of the organ system and high degree of interactions. Specifically, the dynamics of integrin interactions can only be studied on a cellular level. In order to mimick the natural environment in the liver and preserve the cellular polarization [19], [20], cellular clusters of two or more primary rat hepatocytes are used on collagen I or fibronectin immobilized on glass substrates, thereby allowing us to reduce the complexity of the system while still providing the cells with specific signaling cues from certain ECM molecules in order to quantify reactions to osmosensing and osmosignaling events on a cellular level in real time. In addition, we can follow the evolution of contact patches and analyze the membrane bending rigidity and thus the binding strength of the cell-ECM interaction. To specifically visualize the cell-substrate contact area, we utilize a dual-wavelength reflection interference contrast microscope (DW-RICM), also known as a dual-wavelength interference reflection microscope (DW-IRM) [21]. While many microscopy techniques rely on either modification or fixation of samples in order to observe adhesion processes, DW-RICM allows us to quantitatively analyze the contact area of the cells without the need for further sample modifications.

RICM was developed to study adhesion without the need for transfection of cells or fluorescent labeling [22]. It employs monochromatic, polarized light to visualize the interference pattern of an object on a plane glass surface [23]. The RICM image is formed by illuminating the sample from the bottom with parallel light of a distinct wavelength. Each beam of light is reflected partially at the top of the glass surface and at the basal cell membrane. The reflection at an interface with higher optical density, e.g. between the media and the cell membrane leads to a phase shift of the reflected beam of light, which does not occur at an interface with lower optical density such as the glass surface and the media. Thus the light reflected at the glass surface and the cell membrane in close contact with the glass surface interfere destructively with one another leading to a “dark area” in the RICM image. A larger distance between the glass surface and the cell membrane leads to a longer light path of the beam reflected at the cell membrane and thus less destructive or even positive interference with the beam reflected at the glass surface. However, negative interference of the light will not only occur in close contact of the cell with its surface, but also at a height corresponding to a multitude of the wavelength causing the characteristic Newtonian fringes for example in colloidal particles. The resulting RICM intensity is therefore related to the wavelength dependent distance (λ*d) between the glass surface and the reflecting object and can then be used to determine the height at the lateral position x,y of an object using the intensity distribution I:
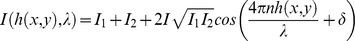
where I is the resultant intensity, I_1_ and I_2_ are the incident light rays, n is the refractive index of the sample and δ is the phase shift of the light reflected from the sample.

DW-RICM using two different wavelengths, which are not multitudes of one another, has so far been successfully applied to improve the precision of height measurements of up to 800 nm and an accuracy of 3 nm in colloidal particles [21]. As the different wavelengths do not interfere with one another the sample can be illuminated and recorded simultaneously using a camera adapter to ensure that only one wavelength is recorded by each of the two cameras enabling fast real time recordings.

However, the interference in RICM is not only determined by the distance of an object to the glass surface, but also by the refractive index of the media and the reflecting object [24]. Thus “dark areas” observed in a single wavelength do not necessarily correspond to close contacts, but might also be caused by accumulation of proteins [25]. As the refractive index is not known in most cell systems as it is not only dependent on membrane composition, but also on the underlying structures such as the actin cortex [26] the fully quantitative height-measuring application of multiwavelength RICM is more suited for well defined colloidal particles [27] or vesicles [28].

Nevertheless, in cell systems DW-RICM still has a great advantage as it improves the accuracy of contact area analysis. Ambiguities of a single wavelength due to the varying refractive indices in different parts of the cell can be eliminated by using the consensus of “dark areas” of two different wavelengths [29]. This allows us to determine a more accurate contact area of any cell and its adaptation over time.

Another advantage of an RICM setup is its use to follow membrane fluctuations and thus membrane bending rigidities of unbound membrane patches by analyzing intensity fluctuations during short timescales in so-calles dynamic RICM (Dy-RICM) [29], [30]. These fluctuations can also be used to find areas of low membrane dynamics indicating tight surface contacts [31].

We use both DW-RICM and Dy-RICM in our analysis of cell-ECM contact change and membrane dynamics during osmotic stimulation of hepatocytes.

## Methods

### RICM Setup

The RICM setup was implemented on an AxioObserver Z1 inverse microscope with a definite focus option using a 63× Plan Neofluar Antiflex oil immersion objective (NA 1.25) (all Zeiss, Göttingen, Germany), a RICM filter cube with a beam splitter (50R/50T VIS) and two polarization filters (AHF, Tübingen, Germany), and a LED light source (Zeiss, Göttingen, Germany) in order to produce coherent, monochromatic light to increase the contrast of the interference pattern. A two camera setup (Orca R2, Hamamatsu, Herrsching, Germany) with a 509 nm beam splitter (AHF, Tübingen, Germany) is used in order to simultaneously image the interference pattern of the 555 nm and the 470 nm wavelength. The sample is kept in a 37°C/5% CO_2_ incubation setup (Pecon, Erbach, Germany) connected to a peristaltic pump (WPI, Berlin, Germany) which allows us to flow aerated and heated Krebs Henseleit (KH) buffer solutions through the sample at a rate of 6 ml/min. This setup allows a fast and easy exchange of KH buffer solutions and also provides a steady pH and temperature in the different buffer solutions. This ensures that the cell volume changes observed are only due to osmotic changes and not pH, temperature or other external factors.

### Cell Culture and Flow Experiments

Hepatocytes were isolated using the technique described previously [32] and cultured on fibrillar collagen I and fibronectin pre-coated coverslips (BD Biocoat, Becton Dickinson, Heidelberg) or self-coated coverslips (incubated with 50 µg/ml overnight at 4°C, both proteins from Sigma-Aldrich, Munich, Germany). Prior to the experiments, the cells were incubated for 24 to 48 h at 37°C/5% CO_2_. In line with previous experiments [14], cells were transferred to 305 mosm/L (normoosmotic) Krebs-Henseleit (KH) buffer 1 hour prior to the experiment to remove all dead cells and debris. The initial relative contact area was recorded with the DW-RICM-setup after the sample was placed in the flow chamber setup and allowed to equilibrate for 20 min under flow. We then stimulated the hepatocytes with 205 mosmol/L (hypoosmotic) KH buffer inducing cell swelling and, after the cells had reached a stable plateau (10 min after hypoosmotic perfusion was started), switched back to the normoosmotic buffer for another 10 min.

### Data Analysis

**Figure 1:**
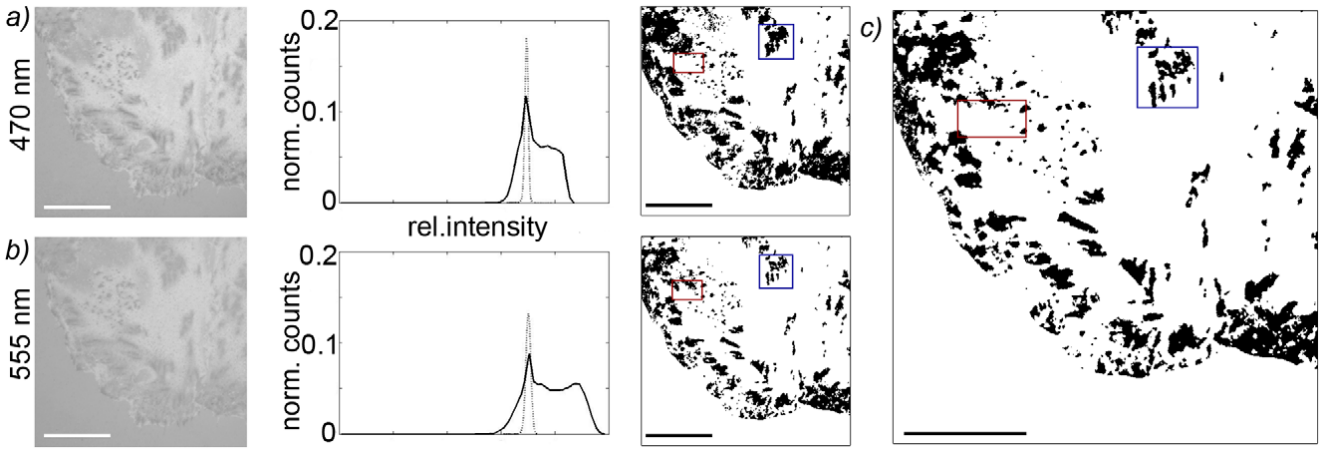
Analysis of DW-RICM images to determine the contact area of hepatocytes. a) and b) show the RICM interferograms for the two different wavelengths used, the histogram of the background intensities (dotted line) and cell intensities (solid line) as well as the contact area determined from the use of the background intensity as a cutoff. For this, the mean intensity I_B_ and standard deviation (σ) of the background were determined and all areas of at least 4×4 pixels with an intensity of less than (I_B_ - ^σ^/_2_) were defined as contact patches. In c) the consensus of the contact patches observed in the two different wavelengths corresponding to the contact area is shown with regions of high variability in the two wavelengths emphasized. Scale bars corresponds to 20 µm.

All data analysis was performed with self-written Matlab routines (Version R2010b). In order to determine the relative contact area of the rat hepatocytes, we first defined the intensity in areas without any cells as background intensity corresponding to the signal intensity of no interference. Next, contact patches in the DW-RICM image of each color were determined separately (Fig. 1 a and b). Due to the negative interference for each wavelength at points of close contact these areas appeared darker than the background intensity. Therefore, the average value (I_B_) and standard deviation (σ) of the complete gauss-fitted background were determined for each image recorded and all areas of at least 4×4 pixels with an intensity of less than (I_B_ - ^σ^/_2_) were defined as contact patches. The consensus of the contact patches observed in the two different wavelengths hence corresponds to the area of the cell in close contact with the glass surface, the contact area (Fig. 1c).

Additionally, we determined the projected cell area over time to quantify changes upon osmotic stimulation. A relative contact area was then introduced to compare different cells: The relative contact area was given by the ratio of the contact area to projected cell area. The values were determined for each recorded image of a time series, values determined for each image in the plateau phases were subsequently averaged to allow for a comparison of all samples.

For membrane dynamics analysis, the amplitude of the intensity fluctuations for a 5 second interval during the equilibration and the plateau phases was analyzed at 10 fps for each 2×2 pixel area of the cells, and the variance was calculated. The average amplitude of these fluctuations was then determined for contact and non-contact regions in each plateau phase.

Data are presented as mean values ± SEM. Statistical significance was assesed using the Mann-Whitney test in Matlab. Statistical significance was assumed for values of p≤0.05.

## Results

### Dynamics of Contact Area Change

**Figure 2:**
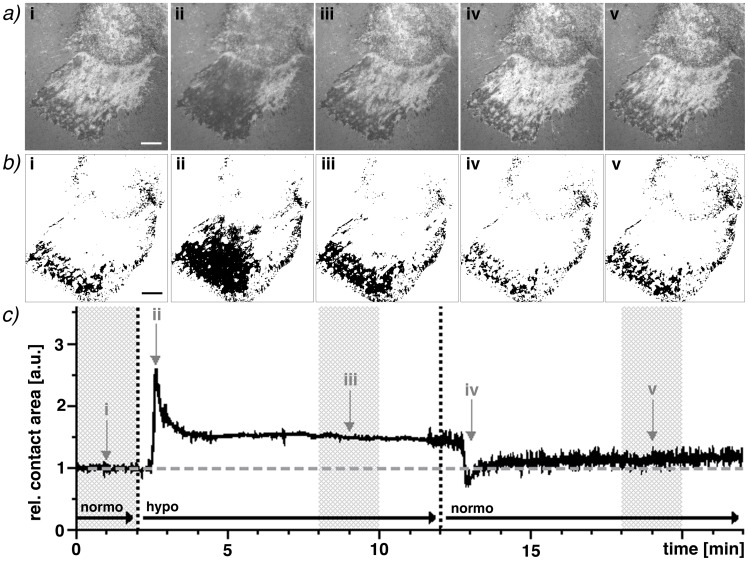
Determination of the changes in contact area during osmotic stimulation of adhered primary hepatocytes. a) The dark patterns in the RICM image of two different wavelengths (470 nm shown here) correspond to cell patches with close surface contact. The original relative contact area (i) changes considerably during hypoosmotic stimulation (ii) and does not return to its original area afterwards (iii). A rapid decrease in relative contact area can be observed after returning to normoosmotic media (iv) followed by the stabilization of the relative contact area (v). b) All areas defined as relative contact areas in the consensus of both wavelengths are represented here as black areas. Images taken at 1 fps in both wavelength simultaneously enable a kinetic analysis: c) The tremendously quick reaction of the hepatocytes to the osmotic stimuli is evident as well as the stable plateaus (compared to the initial relative contact area (i)). The first plateau of this sample (iii) increases by ∼50% and the second (v) by ∼14%). Scale bars correspond to 10 µm.

Continuously recording an RICM interferogram (Fig. 2a) of two separate wavelengths simultaneously enables us to analyze the change in relative contact area (Fig. 2b) over time and to visualize the fast reaction of hepatocytes to a change in osmolarity. This includes the extremely fast swelling and the cell’s rapid regulatory volume decrease within seconds (Fig. 2c). A video of the stimulation can be found in the supplemental information (Video S1).

**Figure 3:**
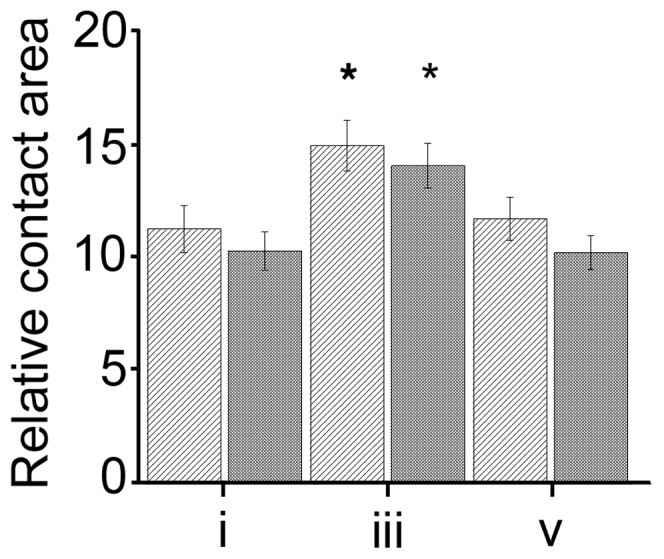
Relative contact area changes in the observed plateau phases. Cells plated on fibronectin (light gray, n = 111) and collagen I (dark gray, n = 98) show different relative contact areas during the calibration phase (plateau i), the plateau phase after the hypoosmolar stimulation (plateau iii) and after the return to normal media (plateau v). (*p≤0.05 with respect to initial relative contact area i), error bars show SEM.

As evident from the interferograms, hepatocytes do not form close contacts to their complete basal side, but are only attached at about 10–15% of their projected cell area (see Fig. 3). The overall relative contact area within the field of view increases after hypoosmolar stimulation. This is not caused by a radial expansion of the cell area, as the projected cell area fluctuates by less than 1.8±0.5% on fibronectin and less than 0.52±0.5% on collagen (See Table S1). Instead, the area of contact between the cell and the coated glass surface increases. It is also interesting to note that the newly formed contacts generally appear around or near areas of previous contact. Thus, this increase occurs by an expansion of the previous contacts rather than through the formation of new contact patches. Similarly, these newly formed patches disappear more readily than the original contacts after a return to the initial buffer.

The cellular reaction to a hypoosmotic stimulation and return to normoosmotic conditions can be divided into three different dynamic regimes: a fast reaction regime, comprising the swelling or shrinking as an immediate response to anisoosmolarities within a few seconds, an intermediate reaction regime, covering the subsequent slower reaction response within tens of seconds, and a stable plateau regime after an equilibrium has been reached.

### Increased Relative Contact Area after Osmotic Stimulation Depending on ECM Composition

One of the important advantages of our DW-RICM setup is its applicability to samples on any glass surface. Thus we are able to compare the change in relative contact area of primary hepatocytes after osmotic stimulation on collagen I as well as on fibronectin coated surfaces (Fig. 3).

On both fibronectin and collagen I surfaces we observe a rapid decrease in the relative contact area within seconds of the initial fast contact increase (ii, see Fig. 2c) and the formation of a stable plateau with an increased relative contact area after a couple of minutes (iii). After the return to normoosmotic conditions (iv), a fast cell shrinkage is observed followed by the cellular counterbalance and the subsequent plateau phase (v).

On collagen I, the cells show a significant increase in their relative contact area from 10.04±0.83% during plateau i to 13.72±0.96% in plateau iii. On fibronectin, the initial relative contact area of 10.99±1.01% is similar to that on collagen I. Cells plated on fibronectin also show a significantly increased relative contact area during plateau iii (14.58±1.1%), making the relative increase in relative contact area slightly higher, but not significantly so, compared to cells plated on collagen I. Once the buffer is exchanged back to normoosmotic buffer and plateau v is reached, cells on collagen I return to the original relative contact area (9.98±0.73%). The same is true for cells plated on fibronectin (11.43±0.94%). Interestingly, the contact patches observed in plateau i show 90% colocalization with the contact patches in plateau v on collagen while only 77% colocalized on fibronectin. For both, this indicates that the change in relative contact area is a reversible process, leaving a majority of initial contact patches unchanged. These significant (p≤0.05) colocalization differences between the two different surface coatings indicate, that the ECM environment plays an important role in the cellular reaction to volume changes. Control experiments with flow but without changes in osmolarity demonstrate no changes in the relative contact area on both surfaces (results not shown).

These observed increases in relative contact area illustrate on a single cell level, that cellular swelling has a direct impact on ECM - contact behavior of hepatocytes. Interestingly, these results also show that there might be subtle differences between differently functionalized surfaces, confirming the biological importance of outside-in signaling of adhesion molecules in osmosignaling events.

### Determination of Membrane Fluctuation Dynamics

**Figure 4:**
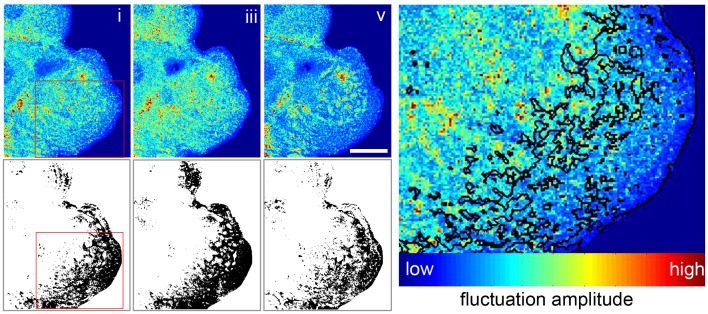
RICM also enables the tracking of changes in the cell’s membrane dynamics. The membrane dynamics can be visualized by the variance of the RICM intensity over 5 seconds at the midpoint of each of the plateaus i, iii, and v. Fluctuations are calculated over a 2×2 pixel area to reduce noise and are visualized in false color with blue being small fluctuations and red larger fluctuations. Corresponding contact images are shown below for comparison. Image on the right shows a magnified area of plateau i with the contact patches marked visualizing lower membrane fluctuations in the areas identified as surface contacts. Scale bar corresponds to 20 µm.

RICM is not only a quick, label-free method for contact area determination, but the same data set can also be used to measure membrane fluctuations on a short time scale. This so-called dynamic RICM (Dy-RICM) indicates membrane patches that are firmly attached to the surface and thus show very low membrane dynamic. More interestingly, it allows us to analyze the bending rigidity of non-bound membrane patches which might be actively adjusted by the cell in response to volume changes. In order to analyze the membrane dynamics, the membrane fluctuation is evaluated at different time points by analyzing the relative intensity of the interferograms during 5 seconds at the midpoint of the respective plateaus. This could be directly correlated to the height changes of the membrane if no variation in the refractive index occurred. Since a reorganization of the internal cell structure is unlikely to occur within the short recording timeframe of 5 seconds within the stable plateau phases, the obtained variance was taken as a measure for membrane height fluctuations. The intensity variance of these time tracks is then depicted to visualize the membrane dynamics of the complete field of view (Fig. 4). The data sets are divided into a contact and non-contact cell area using the contact area analysis described for Fig.1. The relative fluctuations in these areas are then compared for all three plateaus (Tab. 1) in order to gain an insight into the membrane bending rigidity in the different areas at different timepoints.

**Table 1:** Relative membrane fluctuations in contact and non-contact areas.

	Contact			Non-Contact		
	i	iii	v	i	iii	v
collagenn = 36	100±2	103±3	87±2	128±3	130±3	111±2
fibronectinn = 30	93±2*	100±4	86±2	134±4	140±5	118±3
Controls						
collagenn = 12	108±9	92±4	87±7	133±9	112±7	107±6
fibronectinn = 11	99±6	88±6	84±6	142±16	114±11	105±9

Average fluctuations (in percent) according to contact and non-contact areas relative to the fluctuations of the contact area for collagen I in plateau i at the midpoint of each plateau phase (* shows significance with p≤0.05 with respect to collagen, contact area plateau i), as well as the fluctuations at the same timepoints during control experiments where buffer exchange occurred using only normoosmotic buffer.

In order to determine the influence of the flow system on membrane fluctuations, control experiments using only normoosmotic buffers were performed. This data (Table 1) shows that the flow itself causes decreasing membrane fluctuations over time. Such results indicate that changes in the cell membrane or membrane associated structure (such as the cytoskeleton) do occur regardless of the presence of osmotic changes.

The effects of cell volume changes on membrane height fluctuations appears to be similar on the different ECM molecules. For both collagen I and fibronectin the membrane height fluctuations are generally smaller in areas of contact than in non-contact areas, indicating attachment to the surface in regions that have been identified as contact areas by our first data analysis process. Especially the fluctuations in non-contact areas increase slightly in response to the hypoosmotic stimulations (plateau iii) which is different to the normoosmotic control experiments (Table 1). After return to normoosmotic buffer (plateau v) the fluctuations decrease significantly (p≤0.05) with respect to plateau i and plateau iii) on both surface coatings, which is comparable to control experiments. This comparison suggests that the effects observed in plateau iii are due to changes occurring as the cell responds to hypoosmotic stimuli (Table 1).

## Discussion

A challenge with many techniques that have been used to study integrin involvement in osmosensing so far is that they are not suited for *in vitro* cell cluster analysis. However, moving forward to single cell level analysis is necessary to further uncover the fine cellular changes taking place during these vital processes. Previous experiments done on whole liver explants [33]–[35] have shown how liver volume follows a very distinct pattern upon hypoosmotic stimulation. Upon exposing the liver to hypoosmotic perfusate, a fast increase in liver volume occurs which is followed by an equally rapid regulatory volume decrease. After several minutes, a plateau phase is reached and the reactions can be reversed by returning to a normoosmotic perfusate [33]. This pattern is identical to the one we have found in our cluster analysis, except with a slightly different timeframe. Reactions on a cell cluster level seem to occur slightly faster than in the whole liver explant system. This observation underlines the necessity to proceed to cell cluster analysis in order to decrease the complexity of the analyzed system.

Therefore, we decouple the osmotic influences of the ECM and other cell types from the observable hepatocyte reaction and determine the influences of signaling provided by specific ECM molecules by using controllable adhesive surfaces in a DW/Dy-RICM setup. This label-free observation of primary hepatocytes with different underlying substrates during osmotic stimulation allows us to not only record changes in the relative contact area but also to analyze the membrane dynamics in areas of close cell-ECM contacts and for freely fluctuating membrane. This is quite useful, since regions of low height fluctuation recorded by Dy-RICM represent areas with adhered membrane patches [36]–[38]. In contrast to these adhesion areas the flexibility of the membrane in non-contact areas is directly related to the physical properties of the membrane and its underlying structures such as the actin cortex.

We are able to show that the initial attachment depends on the ECM molecule presented to the cells, leading to a significantly lower membrane dynamics and therefore tighter membrane attachment on fibronectin compared to collagen I. Interestingly, these stronger fibronectin adhesions show a higher variability leading to a reorganization of 23% of the contact areas in plateau v, after hypoosmotic stimulation was completed and cells are returned to normoosmotic buffer. Only 10% of the initial contact areas are rearranged on collagen I. Thus, these cell-ECM interactions involve either a variety of integrins or different availability of integrin attachment sites on the immobilized ECM molecules or, most likely, a combination thereof.

After hypoosmotic stimulation and regulatory volume decrease (plateau iii) the contact area of the hepatocytes is significantly increased. But neither the projected cell area nor the local bending rigidity is altered as the membrane fluctuations of the contact areas in plateau iii, after hypoosmotic stimulation, are similar to the fluctuations of initial contact area in plateau i (Fig. 4). This adhesion increase is completely reversed once the cell is returned to a normoosmotic environment. This could be an important indicator for the existence of a signaling mechanism or switch, which only functions when an increase in volume is registered.

This leaves the question why hepatocytes require larger areas of adhesion with the same adhesion strength when there is no change in the projected cell area during hypoosmotic stimulation. Possibly, this is related to the slight increase in membrane height fluctuations in non-contact areas especially compared to the slight decrease in non-stimulated control experiments. Generally, membrane fluctuations of simple membranes are directly related to tension, adhesion and rigidity [39]. As we could observe an increase of the adhesion area and the hepatocytes volume is maintained at a slightly larger volume after swelling [40] leading to an increased tension on the cell membrane we would expect an increase in membrane bending rigidity and thus a decrease in membrane fluctuation in non-contact patches. The fact that we observe a decrease of membrane bending rigidity instead indicates an active change of either the cell cortex or its membrane in direct response to the swelling. These results are also in contrast to studies done on giant unilamellar vesicles, which show an increase in membrane fluctuations i.e. decreased membrane tension when osmotically deflated [41]. This also indicates, that during osmotic stimulation of cells, membrane tension is influenced by more than pure thermal fluctuations and that signaling through cellular volume fluctuations leads to an active cell response.

While the amount of membrane has been shown to increase during hypoosmotic stimulation of neurons [42], a similar increase has been ruled out for hepatocytes in patch clamp experiments [43]. However, these experiments in suspension do not account for the effects of cell adhesion on membrane rearrangement during osmotic signaling. A more likely reason for higher membrane fluctuations after an increased relative contact area due to hypoosmotic stimulation could be an unfolding of membrane from preexisting surface reserves as has been reported in electron microscopy studies of fixed hepatocytes [40] as well as for fibroblasts and lung carcinoma cells [44].

In this study we can follow the active reaction of primary hepatocytes to cellular volume fluctuations in contact with differently coated surfaces. To improve the understanding of the signalling pathways involved, this setup can be further used to study different integrins by e.g. specific inhibition of integrins [14] or by adhesive nanostructured surfaces functionalized with peptides specific to certain integrins with a passivated background to avoid deposition of new ECM molecules [45]–[47]. This will help us to further uncover the role the ECM and its components play in osmosensing and osmosignaling. Additionally, we will be able to study the effect of different osmotic stimuli (e.g. insulin, glutamin) or of specific inhibitors of osmosignaling on adhesion on a single cell level and thus enhance our understanding of their functioning.

With the discovery of cellular volume fluctuations as a signaling tool, many questions about the exact mechanism as well as the sensing and transduction of these signals have been uncovered. So far, the direct link between cellular adhesion and these signaling events has not been directly observed *in vitro*. By studying the relative contact change in cells on differently coated surfaces we are now able to analyze the reaction of a hepatocyte cluster to these osmotic changes and observe not only its surface contact but also its membrane rigidity in real time, non-invasively and label-free.

## Supporting Information

Table S1 **Changes in total cell area during the experiments on the different surface coatings.**
(DOC)

Video S1 **The video shows a hepatocyte cluster during a hypoosmotic stimulation experiment.** The plateau phases used for data analysis are marked along with the buffer changes. The width of the video corresponds to 50 µm.
(M4V)
