# Supplementary material for: Investigating Cell-ECM Contact Changes in Response to Hypoosmotic Stimulation of Hepatocytes In Vivo with DW-RICM
Source: PLoS One. 2012 Oct 26;7(10):e48100. doi: 10.1371/journal.pone.0048100 (PMC3482193; doi:10.1371/journal.pone.0048100)
Supplement: Table S1 — Changes in total cell area during the experiments on the different surface coatings. (DOC) [file pone.0048100.s001.doc]

**Table S1: Changes in adhesion area during stimulation experiments**

| **Surface coating** | **Plateau phase** | **Projected cell area (compared to Plateau i)** | **Standard deviation** |
| --- | --- | --- | --- |
| **Fibronectin** | iii | 101,8 | 0,489 |
|  | v | 99,95 | 0,367 |
| **Collagen I** | iii | 99,99 | 0,533 |
|  | v | 99,48 | 0,538 |

Changes (in percent) in total cell area during the course of the experiments on fibronectin or collagen I.
